# Establishment and analysis of online energy baseline for water purification plants: A case study in Kaohsiung city, Taiwan

**DOI:** 10.1016/j.heliyon.2024.e33981

**Published:** 2024-07-03

**Authors:** Da-Sheng Lee, Shih-Kai Fu, Chih-Wei Lai

**Affiliations:** National Taipei University of Technology Energy and Refrigerating Air Conditioning Engineering, Room 610, College of Mechanical & Electrical Engineering, Integrated Technology Complex, No.1, Sec. 3, Zhongxiao E. Rd., Da'an Dist., Taipei City, 10608, (R.O.C.), Taiwan

**Keywords:** Energy baseline, Regression, Azure, Water purification plants

## Abstract

Water and energy are closely linked and are crucial for national security and economic development. Most water providers prioritise the stability of water supply and aim to reduce energy consumption under the premise of a stable supply. The average energy required to supply water in Taiwan in one of the lowest worldwide. In the Kaohsiung area, the average energy used by a water purification plant to provide 1 m^3^ of water is 0.32 kWh/m^3^, lower than the world average of 0.37 kWh/m^3^. However, the most energy-consuming plant (Weng Park water purification plant) uses eight times as much energy as the least energy-consuming plant (Pingding water purification plant). Most studies focus on the energy required to provide 1 m^3^ of water. This study combined attributes of four plants, such as the amount of energy consumed, quantity of water supplied, purified, and collected, and weather data. These data were used to model energy baselines for water providers. Artificial intelligence was imported into Microsoft Azure machine learning to train the model, which was verified using another Kaohsiung plant and one overseas to establish an online energy baseline modelling system that can be applied in various water purification plants.

## Highlights

• This study used the factory energy baseline to represent the energy baseline of water supply companies.
• Artificial intelligence was imported into Microsoft Azure machine learning to train the model.
• Unlike studies using only regression, this study uses AI methods with various parameters affecting energy consumption.
• Used Linear, Neural Network, and Support Vector Regression to collect annual data from water purification plants.

## 1 Introduction

Water, energy, and food are essential resources required to maintain daily life, reduce poverty, and improve sustainable development (FAO of the United Nations, 2014) [1]. The three resources form a water–energy–food nexus (WEF nexus). Green and Blue Infrastructure (GBI) offers innovative solutions to address the food-water-energy (FWE) nexus. GBI includes features such as urban gardens, green roofs, and wetlands, which contribute to food production, energy conservation, stormwater management, and the purification and collection of rainwater. Implementing GBI can assist urban planners in supporting sustainable development goals [2]. Energy is used in areas of water supply including water pumping, treatment, and distribution and by desalination or reclaimed water plants. Water is used to cool equipment in thermal and nuclear power plants, or to drive hydraulic turbines and generate hydroelectric power. Biomass energy generation also requires water. Food production requires water (such as in rice paddy fields) and energy, such as in the plantation, production, processing, and delivery of food. These resources form the WEF nexus; thus, a single policy that improves water, energy, or food security could affect the other two resources. Studies are now conducted on the competition between these resources as confrontations or conflicts between these resources as a result of rising demand for each. Gleick [3] proposed the energy–water nexus (EWN) to indicate that water is closely linked to energy. Water companies consume large amounts of energy [4] to maintain a stable water supply. The demand for water and its quality continues to increase, resulting in increases in the processes such as water pumping, distribution, and treatment. This in turn increases the demand for energy, with energy used for water supply [5], water distribution [6], the final stage of wastewater treatment [7], wastewater treatment [8], and water heating and cooling [9]. Currently, 7 % of global energy is consumed in the production and distribution of drinking water and in wastewater treatment [10]. For wastewater treatment, various water treatment technologies focusing on energy demand and cost have been developed. Traditional methods such as coagulation and sedimentation are characterized by low energy demand, while advanced membrane technologies, although energy-intensive, achieve higher removal efficiency. The most energy-intensive stage in water treatment is pumping, highlighting the importance of optimizing energy consumption in water treatment plants [11].

The Commonwealth Scientific and Industrial Research Organisation estimates that the world population could increase by 21 % by 2050, thus increasing the demand for water and energy [12]. This study focuses on the relation between water and energy. Water resource management practices are related to the management of water consumption in agriculture, hydroelectric power generation, and other usages. To address the lack of energy, water resource management should exploit new water sources and reduce water consumption. Incorporating renewable energy into water management practices can significantly enhance sustainability, while mitigating global warming and reducing emissions of local and global pollutants. Renewable energy technologies such as solar and wind power can provide energy for water purification and distribution systems, thereby reducing the overall energy consumption and environmental footprint of water utilities [13]. Under the premise of stable water supply, water supply methods that have higher energy consumption should be avoided. Water should be used to maximise hydroelectric power generation, or it should be supplied as cooling water for thermal or nuclear power plants. These principles have been widely applied in water resource management and operations.

The development of Artificial Intelligence (AI) and Machine Learning (ML) technologies has revolutionized water resource management. AI-based systems, including data analytics, regression models, and optimization algorithms, can monitor, manage, and predict water resources more efficiently and accurately. These technologies can optimize water treatment processes, predict potential damages, and enhance the accuracy of sensor-based systems in agriculture [14]. Studies have compared and analysed the energy required to provide 1 m^3^ of water [15] but have rarely conducted energy baseline analyses. Regions may have distinctive water consumption costs due to terrain, water quality, or policies. Therefore, in addition to comparing the water and energy consumption of different regions, the energy baseline of energy required for water supply should also be used understand how energy efficiency can be improved. This project proposes using Microsoft Azure machine learning and artificial intelligence (AI) calculations to calculate the energy baseline for water purification plants and compares it with actual energy consumption. By using a cloud energy baseline modelling system, different regions or different states can model the same baseline, and personnel can revise the energy consumption of water purification plants and set standards to improve energy efficiency.

## 2 Material and methods

### 2.1 Study area

Most reservoirs in Taiwan are located on mountains, and water passes from them to purification plants by gravity. Some areas have cleaner water sources and do not need excessive water treatment, thereby reducing the energy required to supply water.

**Fig. 1:**
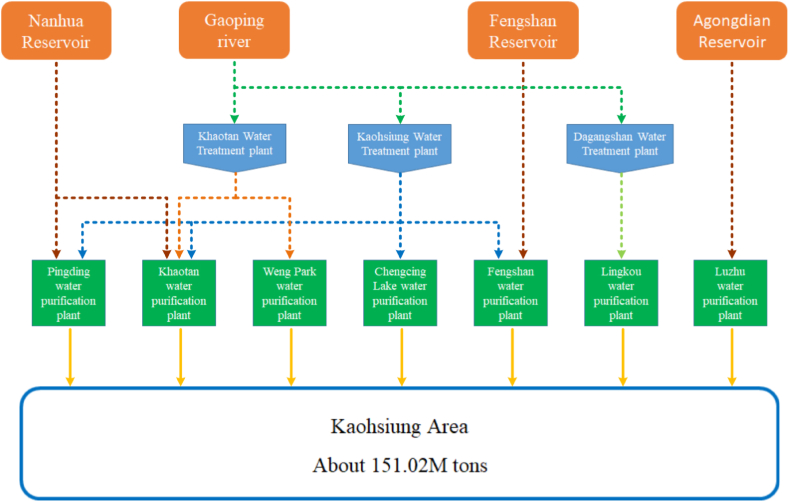
Main water supply systems in Kaohsiung area.

This study used the Kaohsiung area as the main source of data for AI model training. The Gaoping River basin is the largest basin in the Kaohsiung area, and the water supply systems include the Nanhua Reservoir－Jiaxian Weir－Gaoping River Weir connection pipeline project, Agongdian Reservoir system, and Fengshan Reservoir system, as shown in Fig. 1.

The annual water consumption report of Taiwan's Water Resources Agency includes statistics on domestic water consumption, agricultural water consumption, industrial water consumption, and water consumption under other indices. The most recent report indicated that the average total water consumption of southern Taiwan over the previous 10 years was 3.966 billion m^3^; 2.716 billion m^3^ were used for agricultural purposes (68 %), 0.711 billion m^3^ for civil purposes (18 %), and 0.539 billion m^3^ for industrial purposes (14 %). The average domestic water supply, public water supply, and agricultural water consumption in recent years are explained as follows.

Currently, daily water consumption in the Kaohsiung area is 1.515 million m^3^. According to the 2019 Southern Area Water Resource Management Assessment and Evaluation Plan, the projected daily public water consumption for 2031 is 1.865 million m^3^ (Reference [16]).

The Taiwan Water Corporation (seventh branch) is responsible for domestic and public water supply in Kaohsiung. The main water source is river water from the Gaoping River Weir. The average annual public water consumption in the Gaoping River basin in recent years was 0.481–0.531 billion m^3^; the average agricultural water consumption was 0.391–0.538 billion m^3^. Other water sources include the Agongdian Reservoir, the Chengcing Lake Reservoir, the Fengshan Reservoir, and underground water. According to the 2019 reservoir overview data provided by the Water Resources Agency, the Agongdian Reservoir has an effective water capacity of 15.23 million m^3^; the Chengcing Lake Reservoir an effective water capacity of 2.63 million m^3^; and the Fengshan Reservoir an effective water capacity of 7.16 million m^3^ (all capacities measured in 2019). According to the 2019 reservoir operation data provided by the Water Resources Agency, the annual water supply of Gaoping River Weir, the Agongdian Reservoir, the Chengcing Lake Reservoir, and the Fengshan Reservoir is 289.97 million m^3^, 4.579 million m^3^, 119.264 million m^3^, and 99.175 million m^3^ (for industrial purposes), respectively. Again, some water is supplied by underground sources.

### 2.2 Global energy consumption required to provide water

**Fig. 2:**
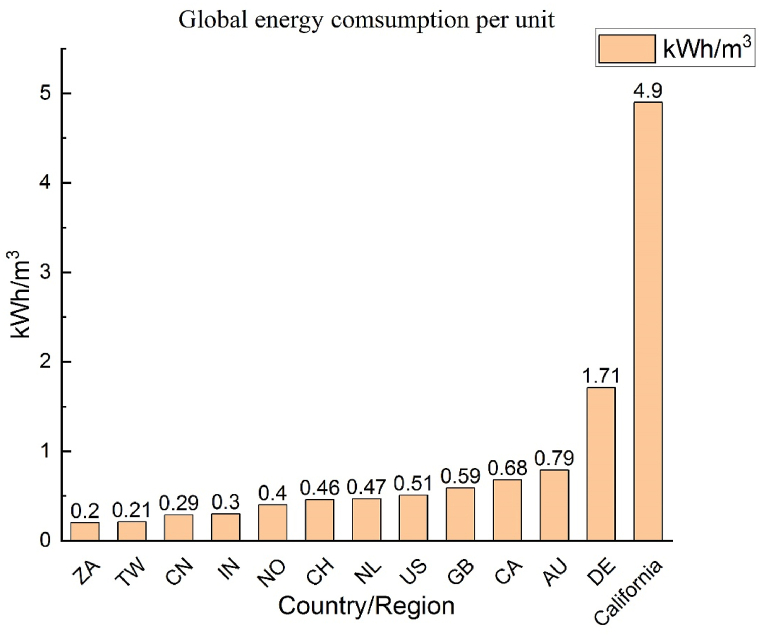
Energy required to provide 1 m^3^ of water in different states [17]. (Country abbreviation:ZA:SOUTH AFRICA, TW: TAIWAN, CN: CHINA, IN: INDIA, NO: NORWAY, CH: SWITZERLAND, NL: NETHERLANDS, US: UNITED STATES, GB: UNITED KINGDOM, CA: CANADA, AU: AUSTRALIA, DE: GERMANY.)

**Fig. 3:**
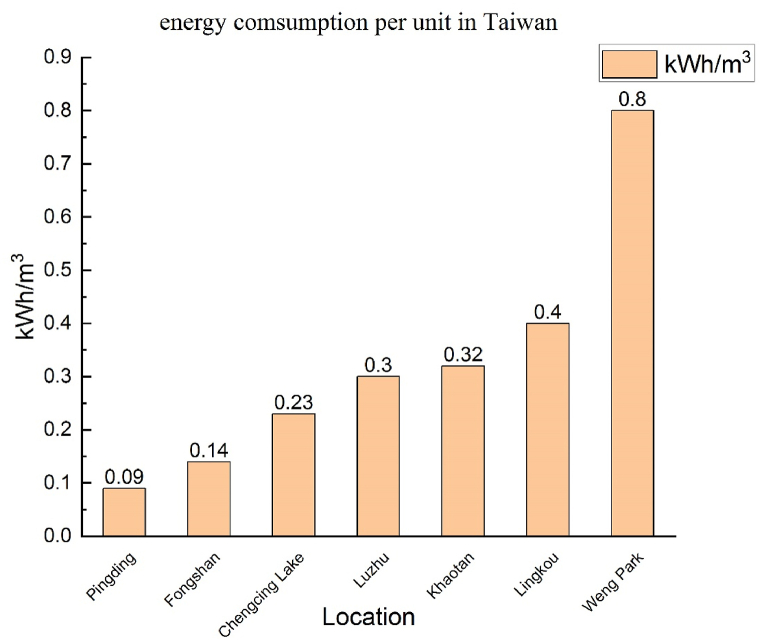
Energy required by water purification plants in Kaohsiung area.

Water supply is crucial to any city. This water is used for agricultural, business, industrial, public, and domestic purposes. The energy consumption of water supply is affected by factors such as elevation, climate, population density, infrastructure, and policy. However, all nations prioritise a stable water supply over the energy required to supply water. According to the United Nations World Water Development Report, the average amount of energy required to provide water from surface water, such as a lake or river, is 0.37 kWh/m^3^. A literature review indicates significant differences in the energy required to provide water in various regions. For instance, the amount of energy needed in California is 4.9 kWh/m³, whereas in German cities, it is 1.71 kWh/m³. In comparison, Asia generally requires less energy to supply water than Europe or North America. For example, India requires 0.3 kWh/m^3^, and Taiwan requires only 0.21 kWh/m^3^. Fig. 2 depicts the energy required to provide water in various states; Taiwan is a top performer. However, if Taiwan is assessed by region or city, the energy required to provide water differs for each. In the Kaohsiung area, for example, the energy consumption of the Pingding water purification plant is only 0.09 kWh/m^3^, whereas that of the Weng Park water purification plant is 0.8 kWh/m^3^, eight times higher than that of the Pingding water purification plant and four times higher than Taiwan's average energy consumption (0.21 kWh/m^3^), as shown in Fig. 3 The reason that the Weng Park water purification plant requires such a large amount of energy is because it provides water to regions located at a higher elevation. Thus, raw water pumping and water distribution systems require additional pressure, resulting in higher energy consumption.

### 2.3 Electricity data

This study collected the annual energy consumption data of five water purification plants in the Kaohsiung area, which included a total of 115 water pumps, 29 backwashing devices, and 24 pieces of sludge dewatering equipment. The energy consumption of these plants is separated into three stages: a water collection stage, a water purification stage, and a water distribution stage. The ratios indicate that each plant uses energy differently. Some plants are located at a higher elevation and can use gravity to collect or supply water; therefore, the energy usage for water collection or distribution for some plants is 0 %, as shown in Table 1.

(a) Energy consumption of Chengcing Lake water purification plant

**Table 1:** Energy consumption of each water purification plant in Kaohsiung area.

Location	Energy consumption		
	Water collection	Water purification	Water distribution
Pingding water purification plant	15.49 %	84.51 %	0 %
Khaotan water purification plant	8.83 %	85.16 %	6.01 %
Fengshan water purification plant	73.7 %	26.3 %	0 %
Luzhu water purification plant	0 %	65.3 %	34.7 %
Chengcing Lake	52.3 %	11.5 %	36.2 %

The Chengcing Lake water purification plant requires relatively more energy for pumping and distribution because it is located at a higher elevation and requires pressure to collect water from reservoirs or water pumping stations, and to supply water to the urban Kaohsiung area. The next-highest energy-consuming equipment is backwashing equipment used in water treatment, particularly when the equipment malfunctions and the water supply method is changed, resulting in unusually large energy consumption.

(b) Energy consumption of Fengshan water purification plant

The Fengshan water purification plant collects water from two water sources. Water for domestic purposes is pressurised and pumped from the Gaoping River Weir water pumping station to the Fengshan water purification plant, whereas water for industrial purposes is collected from the Donggang River. The Fengshan water purification plant is the second largest water purification plant in Kaohsiung area. It has numerous blowers for water pretreatment that are old and inefficient.

(c) Energy consumption of Khaotan water purification plant

The Khaotan water purification plant was designed to treat underground water; however, factors such as ageing deep wells, difficulty in finding sites to dig new wells, and increasing demand for water resulted in the plant constructing additional sedimentation pools and installing sludge dewatering equipment in 1995 to enable the processing of surface water. The three main water sources are surface water of the Gaoping River Weir, subsurface water of the Gaoping River, and deep well water. The plant collects approximately 0.22 million m^3^ of water daily, with energy costs accounting for 30 % of its total costs. The most energy-demanding system is the low-pressure reverse osmosis water purification system.

(d) Energy consumption of Luzhu water purification plant

The Luzhu water purification plant collects raw water from the Agongdian Reservoir. It was built to supply water for Kaohsiung Science Park of the Southern Taiwan Science Park. Any surplus water is provided to northern Kaohsiung as domestic water supply. Its most energy-demanding equipment is its sludge dewatering equipment.

(e) Energy consumption of Pingding water purification plant

The Pingding water purification plant collects water from two water sources: surface water of the Gaoping River Weir and underground water. The plant collects approximately 0.55 million m^3^ of raw water every day, enough to supply the daily water demand of 1.2 million people. The plant generates over two tonnes of sludge annually and uses gravity to distribute water. The plant uses energy mainly for backwashing and wastewater treatment.

### 2.4 Energy baseline

On June 15, 2011, the International Organisation for Standardisation (ISO) published the ISO 50001 energy management systems–requirements with guidance for use standard. The standard requires organisations to establish an energy performance indicator and an energy baseline. An energy performance indicator and an energy baseline are two connected concepts, and ISO Technical Committee 242 established standards to help organisations and certification bodies to establish, use, and maintain energy baselines for companies, as well as to use energy performance indicators to quantify energy efficiency and its changes. Past data are used in fixed-value analysis or regression analysis to model an energy baseline. The purpose of the baseline is to let the user understand the energy efficiency of a facility in a certain period, and to quantify energy efficiency to predict future energy consumption. The user can compare future energy consumption with the energy baseline and adjust energy usage to improve energy efficiency [18].

The establishment of an energy baseline must include the following three items.

1. A linear function that describes the functions of energy use forcing variables.
2. Coefficients in the equations.
3. A coefficient of variation of the root mean square error (CV-RMSE) representing the uncertainty of the model.

Different regression methods can be used to establish energy baseline models, but most build best-fitting regression models using the coefficient of determination (R^2^) and CV-RMSE. The American Society of Heating, Refrigerating and Air-Conditioning Engineers (ASHRAE) guidelines 14–2002 and 14–2014 state that the ASHRAE Guideline 14, Federal Energy Management Program (FEMP), and International Performance Measurement and Verification Protocol (IPMVP) all provide reliable definitions of uncertainty. The Federal Energy Management Program (FEMP) enhances energy efficiency in U.S. government agencies by providing tools and guidance. The International Performance Measurement and Verification Protocol (IPMVP) is a global standard for assessing energy efficiency projects. Together, they ensure credible and transparent energy-saving efforts in federal agencies.

The main indices for uncertainty are normalised mean bias error (NMBE), CV-RMSE, and the coefficient of determination. These coefficients derive from mean bias error (MBE) and are used to propose recommended CV-RMSE and NMBE, as shown in Table 2 [[19], [20], [21]].

**Table 2:** Calibration standards.

Calibration standard	Type of data			
	Index	FEMP	ASHRAE Guideline 14	IPMVP
Monthly standard	NMBE	±5	±5	±20
	CV-RMSE	15	15	
Hourly standard	NMBE	±10	±10	±5
	CV-RMSE	30	30	20
Suggested model	R^2^	–	>0.75	>0.75

MBE is the average error of samples. It is used to indicate the relationship between simulated data and the regression line of a sample. If the MBE is positive, then the measured data are underpredicted. A negative MBE, by contrast, signifies overprediction. However, with this index, cancellation error can affect MBE. Once the model is calibrated, the regression line of samples is close to the regression line of simulations, thus enlarging the effects of the cancellation error. The expected outcome ($\hat{y_{i}}$) and the actual outcome ($y_{i}$). The calculation of MBE is as follows:

(1) $$\text{MBE}=\frac{\sum_{i=1}^{n}\left(y_{i}-\hat{y_{i}}\right)}{n}$$

NMBE is a normalisation of MBE and is used to scale it for comparison. NMBE divides MBE by the average of the measured values to quantify MBE and calculate the difference between measured and predicted values. Similar to MBE, a positive NMBE signifies underprediction, and a negative NMBE signifies overprediction. NMBE also cancels out error; thus, using this index alone is not recommended. The calculation of NMBE is as follows. In the equation, *p* is the number of adjustable model parameters, which is suggested to be zero for calibration.

(2) $$\text{NMBE}=\frac{\sum_{i=1}^{n}\left(y_{i}-\hat{y_{i}}\right)}{\left(n-p\right)∙\bar{y}}\times 100\left(\%\right)$$

CV-RMSE is used to determine how the error between the measured and the predicted value changes. It represents the model's ability to use simulated data to reflect overall load changes. In this case, the value of *p* is suggested to be one. CV-RMSE is not affected by cancellation error. Its calculation is follows:

(3) $$\text{CV}-\text{RMSE}=\frac{1}{\bar{y}}\sqrt{\frac{\sum_{i-1}^{n}{\left(y_{i}-\hat{y_{i}}\right)}^{2}}{\left(n-p\right)}}\times 100\left(\%\right)$$

R^2^ is another common statistical index used to measure the uncertainty of a model. It measures the distance of predicted values from the regression line of measured values. The range of R^2^ is between zero and one, with a higher value indicating that the predicted values are closer to the measured values. The calibration value is recommended to be 0.75 or higher. The calculation of R^2^ is as follows:

(4) $$R^{2}={\left(\frac{n∙\sum_{i=1}^{n}y_{i}∙\hat{y_{i}}-\sum_{i=1}^{n}y_{i}∙\sum_{i=1}^{n}\hat{y_{i}}}{\sqrt{n∙\sum_{i=1}^{n}{y_{i}}^{2}-{\left(\sum_{i=1}^{n}y_{i}\right)}^{2}∙n∙\sum_{i=1}^{n}{\hat{y_{i}}}^{2}-{\left(\sum_{i=1}^{n}\hat{y_{i}}\right)}^{2}}}\right)}^{2}$$

This study used linear regression and neural network regression (NNR) as training methods. The data of four Taiwanese water purification plants were adopted to train the models, and the data of one Taiwanese and one Chinese water purification plant were used to verify the models.

#### 2.4.1 Linear regression

Linear regression predicts the linear relation between dependent variables and independent variables. Its calculation is follows [22]:

(5) $$\hat{y_{i}}=a_{0}x_{i}+a_{1}$$

To find the optimal solution, the residual error ($\epsilon_{i}$) between the expected outcome ($\hat{y_{i}}$) and the actual outcome ($y_{i}$) should be as small as possible.

(6) $$\epsilon_{i}=\hat{y_{i}}-y_{i}$$

The sum of squared residuals (SSR) is calculated by squaring each residual and then adding them up.

(7) $$\text{SSR}=\sum_{i=1}^{n}{\epsilon_{i}}^{2}=\sum_{i=1}^{n}{\left(\hat{y_{i}}-y_{i}\right)}^{2}$$

By adjusting two parameters, $a_{0}$ and $a_{1}$, in equation 2.1, the optimal SSR can be obtained. However, adjusting the two parameters directly is not efficient. Therefore, $a_{0}$ and $a_{1}$ are partially differentiated, and extreme values of the function are obtained by setting the derivative as zero.

(8) $$a_{1}=\bar{y}-a_{0}\bar{x}$$

(9) $$a_{0}=\frac{\sum_{i=1}^{n}\left(y_{i}-\bar{y}\right)\left(x_{i}-\bar{x}\right)}{\sum_{i=1}^{n}{\left(x_{i}-\bar{x}\right)}^{2}}$$

The gradient of the optimal outcome is $a_{0}$. When $a_{0}$ is greater than zero, $\hat{y_{i}}$ increases as $x_{i}$ increases; when $a_{0}$ is less than zero, $\hat{y_{i}}$ decreases as $x_{i}$ decreases. The intercept of the optimal outcome is $a_{1}$. When *x* is zero, $a_{1}$ is equal to the average of the variable $\bar{y}$.

#### 2.4.2 Neural network regression

NNR is constructed from multiple neurons (or perceptrons). A neuron has an input end and an output end. The output end is the value of input end $x_{n}$ multiplied by each weight${\;w}_{n}$. The neurons can be classified as single-input or multiple-input neurons. The calculations are as follows [23,24]:

$$y=f\;\left(x_{1}w_{1}+x_{2}w_{2}+x_{3}w_{3}+\ldots +x_{n}w_{n}\right)$$

(10) $$=f\;\left(\sum x_{n}w_{n}\right)$$

A multilayer perceptron (MLP) is formed from multiple perceptrons. The input layer is connected to hidden layers, which are then connected to the output layer.

An MLP is a forward propagation neural network. The calculations between the input layer and the hidden layer are as follows: a value $x_{n}$ is inputted into the input layer and weighted by the value $w_{i}$. Then, it is changed by the activation function $f_{1}$ and becomes output $h_{n}$ of the hidden layer. The calculations between the hidden layer and the output layer are as follows: value $h_{n}$ of the hidden layer is weighted by the value ${\;w}_{j}$; then, it is transformed by the activation function $f_{2}$ and becomes output $y_{n}$ of the output layer.

The values from the input end to the hidden layer are ${\;S}_{j}$, *j=1, 2 …*; $v_{ij}$ is the weight of the *i*th input to the *j*th hidden node. The calculation is as follows:

(11) $$s_{j}=\sum_{n=0}^{i}w_{nj}x_{n}$$

Using activation function $f_{1}$ of the hidden layer, the output of the hidden node is $h_{j}$, as shown in the following equation:

(12) $$h_{j}=f_{1}\left(s_{j}\right)$$

The value from the hidden layer to the output end is $z_{k}\text{,}$ *k=1,2 …*; $w_{jk}$ is the weight of the *j*th hidden node to the *k*th output. The calculation is as follows:

(13) $$z_{k}=\sum_{n=1}^{j}w_{nk}h_{n}$$

Using activation function $f_{2}$ of the output layer, the predicted output value is $\hat{y_{j}}$, as shown in the following equation:

(14) $$\hat{y_{j}}=f_{2}\left(z_{k}\right)$$

After the output value is calculated, the machine compares the output value with the actual value through backpropagation. Typically, the cost function is used to calculate the mean squared error. An accurate prediction would result in a smaller error, whereas an inaccurate prediction would result in a larger error. When the error is larger, the machine continues its learning until the error is smaller.

$x_{i}$ is the input value of the *i*th set of data; its output value is

(15) $$y_{k}=\left[\begin{array}{c} y_{1}^{i}\\ \vdots \\ y_{k}^{i} \end{array}\right]$$

The mean squared error of $y_{k}$ is

(16) $${MSE}^{i}=\frac{1}{k}\sum_{n=1}^{k}{\left(\hat{y_{n}^{i}}-y_{n}^{i}\right)}^{2}$$

To minimise the mean squared error, the min{MSE} can be found by setting the derivative equal to zero and using gradient descent to find the optimal outcome. The calculations are as follows:

(17) {MSE}wij,wjkmin=min{∑i=1lMSEi}

(18) $$\frac{\partial E}{{\partial w}_{ij}}=0,\;\frac{\partial E}{{\partial w}_{jk}}=0$$

(19) $${w_{ij}}^{\prime }=w_{ij}-\eta \Delta w_{ij}$$

(20) $${w_{jk}}^{\prime }=w_{jk}-\eta \Delta w_{jk}$$

η is the learning rate.

(21) $$\Delta w_{jk}=\frac{\partial MSE}{\partial w_{jk}}=\frac{\partial \sum_{i=0}^{n}MSE^{i}}{\partial w_{jk}}=\sum_{i=0}^{n}\frac{\partial {MSE}^{i}}{\partial w_{jk}}$$

The following four equations are combined into the final equation:

(22) $$\frac{\partial {MSE}^{i}}{\partial w_{jk}}=\frac{\partial MSE^{i}}{\partial z_{k}}\times \frac{\partial z_{k}}{\partial w_{jk}}$$

(23) $$\frac{\partial {MSE}^{i}}{\partial z_{k}}=\frac{\partial \frac{1}{2}\sum_{k=0}^{m}{\left({\hat{y_{k}}}^{i}-{y_{k}}^{i}\right)}^{2}}{\partial z_{k}}=\frac{\partial \frac{1}{2}\sum_{k=0}^{m}{\left(f_{2}{z_{k}}^{i}-{y_{k}}^{i}\right)}^{2}}{\partial z_{k}}=\sum_{k=0}^{m}\left({\hat{y_{k}}}^{i}-{y_{k}}^{i}\right){f_{2}}^{\prime }\left({z_{k}}^{i}\right)$$

(24) $$\frac{\partial z_{k}}{\partial w_{jk}}=\frac{\partial \sum_{j=1}^{k}w_{jk}h_{j}}{\partial w_{jk}}=h_{j}$$

The final equation is

(25) $$\frac{\partial {MSE}^{i}}{\partial w_{jk}}=\sum_{k=0}^{m}\left({\hat{y_{k}}}^{i}-{y_{k}}^{i}\right){f_{2}}^{\prime }\left({z_{k}}^{i}\right)\times h_{j}$$

(26) $$\Delta w_{ij}=\frac{\partial MSE}{\partial w_{ij}}=\frac{\partial \sum_{i=0}^{n}MSE^{i}}{\partial w_{ij}}=\sum_{i=0}^{n}\frac{\partial {MSE}^{i}}{\partial w_{ij}}$$

(27) $$\frac{\partial {MSE}^{i}}{\partial w_{ij}}=\frac{\partial MSE^{i}}{\partial s_{j}}\times \frac{\partial s_{j}}{\partial w_{ij}}=\frac{\partial MSE^{i}}{\partial z_{k}}\times \frac{\partial z_{k}}{\partial s_{j}}\times \frac{\partial s_{j}}{\partial w_{ij}}$$

$$\frac{\partial {MSE}^{i}}{\partial z_{k}}\;\text{has}\;\text{been}\;\text{proven}\;\text{earlier}\;\text{in}\;\text{this}\;\;\text{paper.}$$

(28) $$\frac{\partial z_{k}}{\partial s_{j}}=\frac{\partial \sum_{j=1}^{m}w_{\text{jk}}f_{1}s_{j}}{\partial s_{j}}=w_{\text{jk}}{f_{1}}^{\prime }\left({s_{j}}^{i}\right)$$

(29) $$\frac{\partial s_{j}}{\partial w_{ij}}=\frac{\partial \sum_{i=1}^{d}w_{jk}x_{i}}{\partial w_{ij}}=x_{i}$$

The aforementioned equations are combined to form this equation:

(30) $$\frac{\partial {\text{MSE}}^{i}}{\partial w_{\text{ij}}}=\sum_{k=0}^{m}\left({\hat{y_{k}}}^{i}-{y_{k}}^{i}\right){f_{2}}^{\prime }{z_{k}}^{i}\times w_{\text{jk}}{f_{1}}^{\prime }\left({s_{j}}^{i}\right)\times x_{i}$$

The final equation is

(31) $$\Delta w_{\text{jk}}=\sum_{i=0}^{n}\frac{\partial \text{MS}E^{i}}{\partial w_{\text{jk}}}=\sum_{i=0}^{n}\sum_{k=0}^{m}\left({\hat{y_{k}}}^{i}-{y_{k}}^{i}\right){f_{2}}^{\prime }\left({z_{k}}^{i}\right)\times h_{j}$$

(32) $$\Delta w_{\text{ij}}=\sum_{i=0}^{n}\frac{\partial {\text{MSE}}^{i}}{\partial w_{\text{ij}}}=\sum_{i=0}^{n}\sum_{k=0}^{m}\left({\hat{y_{k}}}^{i}-{y_{k}}^{i}\right){f_{2}}^{\prime }\left({z_{k}}^{i}\right)\times w_{\text{jk}}{f_{1}}^{\prime }\left({s_{j}}^{i}\right)\times x_{i}$$

Backpropagation computes the predicted value from the output end to the hidden layer, and then from the hidden layer to the input end, in order to find the optimal solution.

#### 2.4.3 Support Vector Regression

**Fig. 4:**
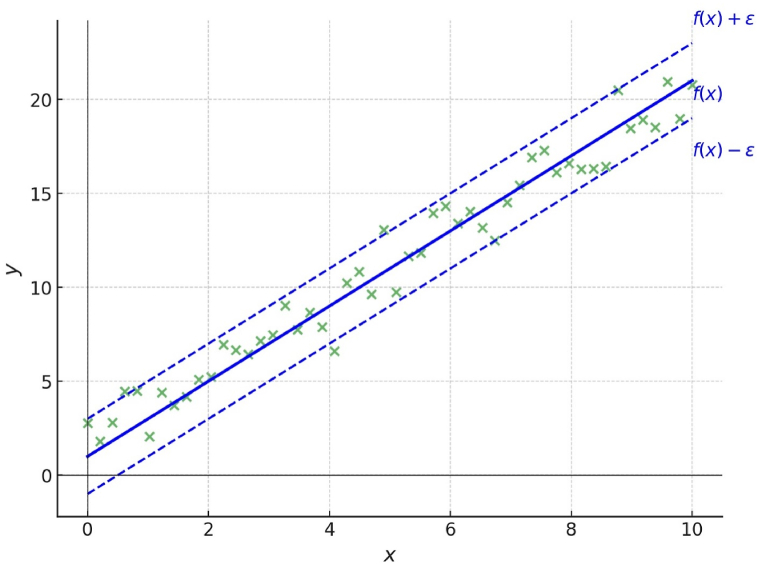
Support Vector Regression (SVR) schematic diagram.

Support Vector Regression (SVR) is a regression analysis method derived from the concept of Support Vector Machines (SVM). SVR maps the training data from the input space to a higher-dimensional feature space using a function, and then constructs a separating hyperplane with maximum margin in the feature space [25]. Both SVR and SVM use a linear kernel function for regression analysis, but if the error is within the tolerance range (ε), it is considered a correct prediction, as shown in Fig. 4 [26].

The basic formula for SVR is shown in Equation (33) [27], where *x* is the input,

***ω*** is the normal vector, (*x*) is the kernel function that maps the input from the low-dimensional space to the high-dimensional space, and *b* is the threshold.

(33) f(x)=(ω∙φ(x))+b

The optimization problem formula for SVR is shown in Equation (34), where *C* is the regularization constant and L(.) is the loss function.

(34) $$\min\frac{1}{2}{\left\|\omega \right\|}^{2}+C\sum_{i=1}^{n}L\left(y\left(i\right),f\left(x\left(i\right),\omega \right)\right)$$

The formula for the loss function (.) is shown in Equation (35), where (*i*) and ((*i*)) are the actual value and predicted value at time *i* respectively, and *ɛ* is the margin generated by the SVR function.

(35) $$L\left(.\right)=\left\{ \begin{array}{c} \left|y\left(i\right)-f\left(x\left(i\right),\omega \right)\right|-\epsilon ,if\;|y\left(i\right)-f(x\left(i\right)\\ 0,otherwise \end{array}\right.$$

#### 2.4.4 Pearson correlation coefficient

There are many types of input parameters for training power consumption prediction models for water purification plants, but not every type of data has an absolute influence on power consumption. The types of data include: 1. Electricity costs generated by power consumption; 2. Site monitoring data: inflow, purified water volume, water supply volume, water supply pressure; 3. Historical data from the Central Weather Bureau (CWB): temperature, humidity, and rainfall.

When faced with a situation where there are many types of input data and it is necessary to filter out the data types most highly correlated with power consumption prediction, Quoc-Thang Phan et al. used the Pearson correlation coefficient (PCC) to measure the relationship between input variables and the target variable (predicted power consumption) [28]. However, this analysis method is only suitable for time series data of continuous input and target variables, with correlation coefficients ranging from 1 to −1. PCC is mainly used to calculate the standard deviation between input variables and the target variable. The coefficient can be used to determine the strength of the relationship between the two. When the coefficient is between 0 and 1, it indicates that as the input variable increases, the target variable also increases. Conversely, when the coefficient is between 0 and -1, it indicates that as the input variable increases, the target variable decreases. A coefficient of 0 means there is no linear relationship between the input variable and the target variable. The PCC formula is given by equation (36) [29]:

(36) $$\gamma_{xy}=\frac{\sum_{i=1}^{n}\left(x_{i}-\bar{x}\right)-\left(y_{i}-\bar{y}\right)}{\sqrt{{\sum_{i=1}^{n}\left(x_{i}-\bar{x}\right)}^{2}}\sqrt{{\sum_{i=1}^{n}\left(y_{i}-\bar{y}\right)}^{2}}}$$

**Fig. 5:**
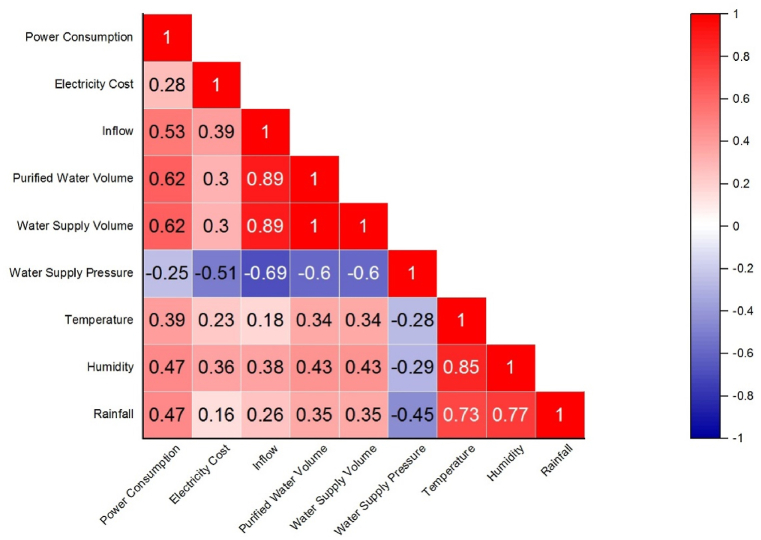
Input training parameter correlation matrix.

According to the correlation matrix plotted using PCC, as shown in Fig. 5, the correlation between Power Consumption (target variable) and Water Supply Pressure is relatively low. Therefore, in subsequent model training, this input variable will be removed, and the remaining data will be used as input variables.

### 2.5 Microsoft Azure machine learning

Most energy baseline calculations use linear regression, but the results are typically not satisfactory. Therefore, this study chose to use linear regression, NNR, and SVR to train the models. The Microsoft Azure machine learning AI platform, with which cloud services can be used to build a program, was employed. It also supports R and Python, and includes various simulation tools and algorithms.

**Fig. 6:**
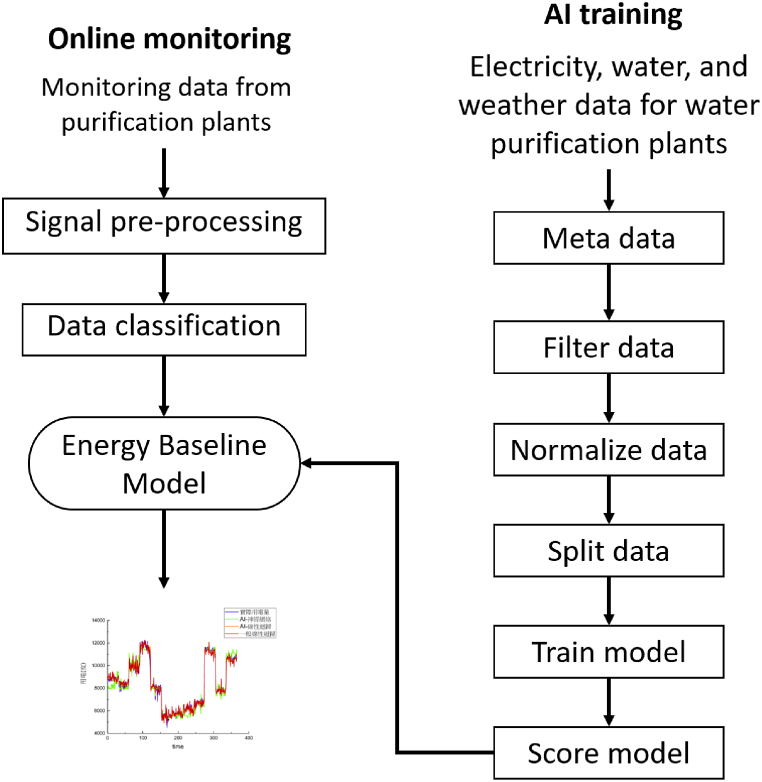
Study flowchart.

Fig. 6is the study flowchart. First, the energy, water, and weather data of the water purification plants were filtered by the program to remove inapplicable data such as noise in the energy data or data on abnormal equipment activity. Although some noise was removed, the units and property of the data remained drastically different. Thus, the data were normalised before training. After these operations were completed, the data were used for AI training. The training used NMBE, CV-RMSE, and the coefficient of determination as uncertainty indices. The AI-trained energy baseline can be placed on a cloud platform, and water purification plants elsewhere can go online, model a corresponding energy baseline, and establish energy-saving goals and regulations.

## 3 Results and discussion

### 3.1 Modelling the energy baseline

This study compared common energy baseline modelling methods, namely AI training using linear regression, NNR, and SVR. Areas of improvement could be due to old equipment, human operations, or policies. A comparison between the actual power consumption and energy baseline cannot determine which method is more suitable for energy baseline modelling because the baseline is used to predict how energy efficiency can be improved. Most energy baselines are modelled through regression analysis, as its calculations are simpler, and it does not require additional data processing and is not limited to complex conditions. The results shown in Table 3 were compared with regulations in ASHRAE guidelines 14–2002 and 14–2014. The R^2^ values of the Fengshan and Khaotan water purification plants were lower than 0.75 and thus did not comply with the regulations. Only the Chengcing Lake and Luzhu plants had R^2^ values larger than 0.75. The NMBE and CV-RMSE values of the plants complied with the regulations.

**Table. 3:** Actual energy consumption and energy baseline of four water purification plants.

	result		
Methods	NMBE	CV-RMSE	R2
Chengcing Lake			
Linear Regression	0.0706	2.0121	0.7879
Neural Network Regression	4.4841	22.9027	0.8479
Support Vector Regression	0.0521	1.7806	0.8436
Fengshan			
Linear Regression	0.0450	1.5101	0.7130
Neural Network Regression	−2.1624	26.9146	0.7944
Support Vector Regression	0.0596	1.2590	0.7926
Khaotan			
Linear Regression	0.0384	0.8795	0.7151
Neural Network Regression	0.8001	0.7561	0.7728
Support Vector Regression	0.0024	0.7987	0.7677
Luzhu			
Linear Regression	0.0771	3.9770	0.9153
Neural Network Regression	−0.2809	6.6911	0.9883
Support Vector Regression	0.0002	2.8330	0.9868

In addition to linear regression, this study used NNR and SVR to model energy baselines. However, NNR and SVR requires additional AI training and large amounts of data; therefore, the annual data of the Fengshan, Chengcing Lake, Khaotan, and Luzhu plants were collected and normalised for AI training. The data included the amount of energy consumed, the quantity of water supplied, purified, and collected, and weather data. The trained model was used to model the energy baseline for the four plants. As Table 3 indicates, the R^2^ value of each was greater than the 0.75 recommended by ASHRAE guidelines 14–2002 and 14–2014. Even though NMBE and CV-RMSE were larger relative to the results of the regression analysis, their values still complied with the ASHRAE regulations.

**Fig. 7:**
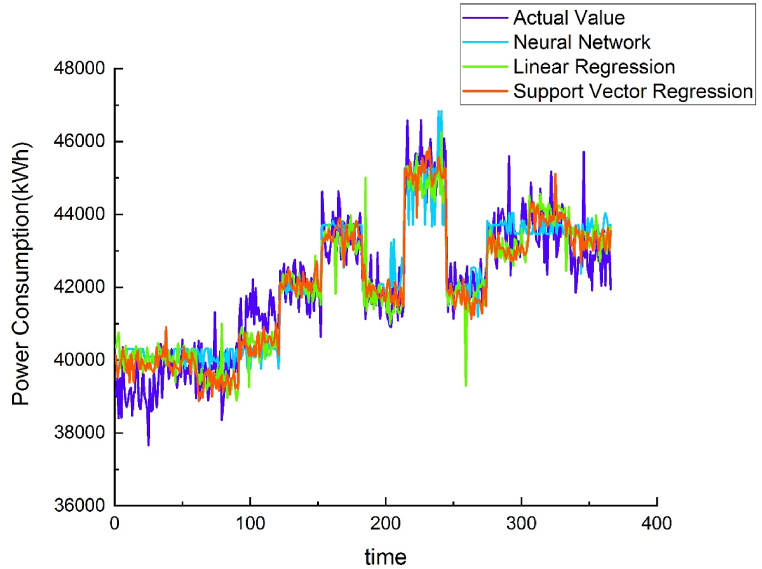
Chengcing Lake water treatment plant energy baseline modeling comparison.

**Fig. 8:**
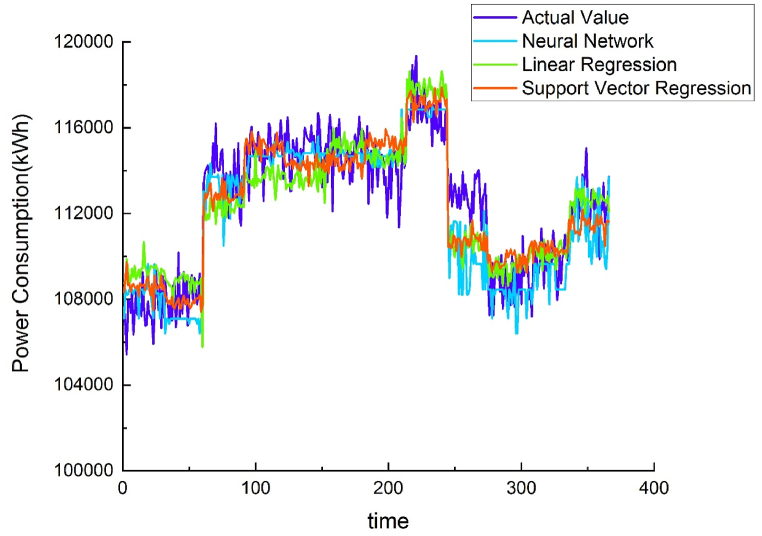
Fengshan water treatment plant energy baseline modeling comparison.

**Fig. 9:**
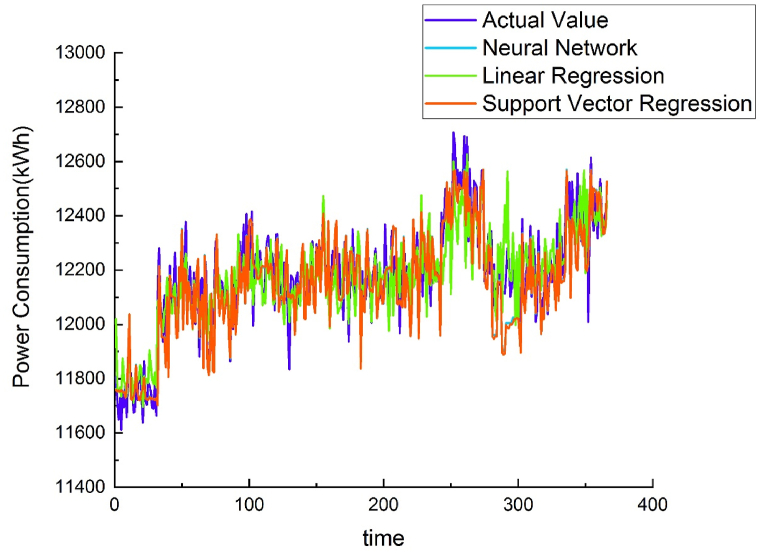
Khaotan water treatment plant energy baseline modeling comparison.

**Fig. 10:**
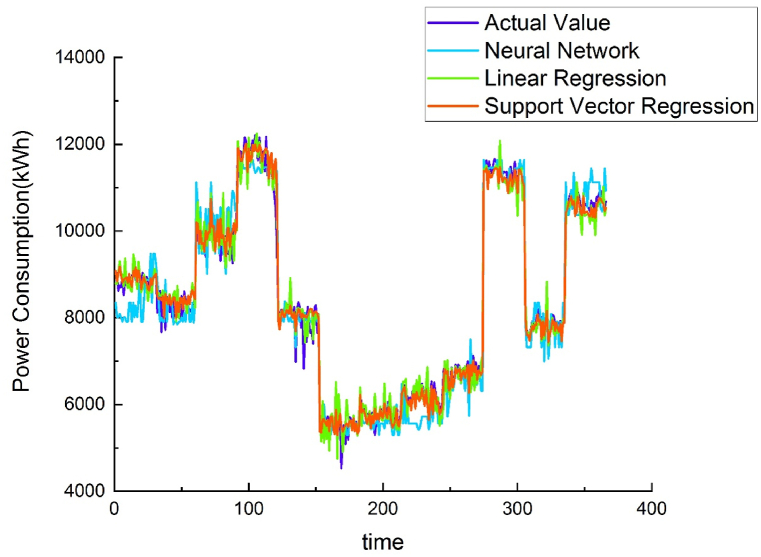
Luzhu water treatment plant energy baseline modeling comparison.

Using different methods to model the energy baseline of each water purification plant demonstrated that the results of the NNR method were similar to those of the SVR method, but NNR R^2^ values were greater than SVR. Both methods were superior to linear regression. Therefore, the AI-trained model was placed on the cloud, and data from other water purification plants were used to verify the applicability of the energy baseline model. (see Fig. 7, Fig. 8, Fig. 9, Fig. 10).

### 3.2 Applications of energy baseline

The previous section mentioned using the data of four water purification plants to train an energy baseline model and set it on the cloud. However, not all water purification plants have abundant data. Some plants have only singular statistics such as water or energy consumption. Linear regression requires large amounts of detailed data for accurate analysis. Fewer data generate less accurate results, and recalculations will be required for different sites. The energy baseline model developed in this study was set on the cloud; therefore, it can be used anywhere. It also does not require many types of data. To verify it, the energy consumption data of the Pingding plant was used. Table 4 presents the results; the NMBE, CV-RMSE, and R^2^ values all complied with ASHRAE regulations.

**Table 4:** Results of energy baseline verification using neural network regression.

Region-Methods	NMBE	CV-RMSE	R2
Pingding Neural Network Regression	7.6001	19.9193	0.7940
China Neural Network Regression	0.2237	4.1833	0.8030

As using the data of another water purification plant in Kaohsiung is less objective, in addition to the data of the Pingding plant, the data of a water purification plant in China were also used for verification [30]. This plant is located at a different region and has fewer types of data, making it suitable for verification. Table 4 contains the results. The NMBE, CV-RMSE, and R^2^ values all complied with ASHRAE regulations. Therefore, the energy baseline model is applicable for plants in different regions.

## 4 Conclusions and future prospect

Traditional water purification plants prioritise a stable water supply over energy-saving measures. Where plants have energy-saving measures, they are generally measures for a single piece of equipment and not for the entire plant. Most studies have researched the energy required to provide 1 m^3^ of water; however, the energy consumption of water purification plants is often related to other factors, such as the amount of energy consumed, the quantity of water purified, supplied, and collected, and weather. This is similar to the energy consumption of factories; numerous factors affect their energy consumption. Therefore, this study used the energy baseline, which is often used in factories, to represent the energy baseline of water companies providing water supply. This can improve energy efficiency, and the energy baseline model trained by AI can be built on the cloud and used in other scenarios. Most energy baselines are modelled with regression analysis, but varying locations and the lack of data can result in inadequate NMBE, CV-RMSE, and R^2^ values for the baseline. Therefore, this study used the NNR and SVR methods and collected annual data from the Fengshan, Chengcing Lake, Khaotan, and Luzhu water purification plants. The data included the amount of energy consumed, the quantity of water purified, supplied, and collected, and weather data. The data were used with AI to train the energy baseline model. The results indicated that the energy baseline modelled by NNR had R^2^ values superior to those for linear regression and SVR, and the NMBE, CV-RMSE, and R^2^ values all complied with ASHRAE regulations. The model was also verified using the data of two water purification plants, one in Taiwan and the other in China. The results indicated that these plants had R^2^ values greater than 0.75, and their NMBE, CV-RMSE, and R^2^ values complied with the regulations of ASHRAE Guideline 14, the FEMP, and the IPMVP, which signifies that this modelling system can be applied in different scenarios.

**Table:** Symbols and Definitions

Symbol	Definition
MBE	Mean Bias Error
NMBE	Normalised Mean Bias Error
CV-RMSE	Coefficient of Variation of the Root Mean Square Error
R^2^	Coefficient of Determination
$\hat{y_{i}}$	Predicted value
$y_{i}$	Measured value
*n*	Number of observations
*p*	Number of adjustable model parameters (suggested to be zero for calibration)
$\epsilon_{i}$	Residual error between the expected outcome and the actual outcome
SSR	Sum of Squared Residuals
$a_{0}$	Intercept of the linear regression model
$a_{1}$	Slope of the linear regression model
$x_{i}$	Independent variable
*f*	Activation function in neural network regression
$x_{n}$	Input value for the *n*-th neuron in neural network regression
$w_{n}$	Weight for the *n*-th neuron in neural network regression
$h_{j}$	Output value of the hidden layer in neural network regression
$s_{j}$	Value from the input end to the hidden layer
$v_{ij}$	Weight of the *i*-th input to the *j*-th hidden node
$z_{k}$	Value from the hidden layer to the output end
$w_{\text{jk}}$	Weight of the *j*-th hidden node to the *k*-th output node
*η*	Learning rate in neural network regression
$\Delta w_{\text{jk}}$	Change in weight from the *j*-th hidden node to the *k*-th output node
$\Delta w_{ij}$	Change in weight from the *i*-th input to the *j*-th hidden node
*MSE*	Mean Squared Error
∂	Partial derivative
min	Minimization function

## Data availability statement

● The data associated with this study has not been deposited into a publicly available repository.
● The authors confirm that the data supporting the findings of this study are available within the article. Raw data that support the findings of this study are available from the corresponding author, upon reasonable request.

## CRediT authorship contribution statement

**Da-Sheng Lee:** Writing – review & editing, Writing – original draft. **Shih-Kai Fu:** Writing – review & editing, Writing – original draft, Software, Methodology, Data curation, Conceptualization. **Chih-Wei Lai:** Validation, Data curation.

## Declaration of competing interest

The authors declare that they have no known competing financial interests or personal relationships that could have appeared to influence the work reported in this paper.

## References

[1] Li Mo, Fu Qiang, Singh Vijay P., Liu Dong, Li Tianxiao (May. 2019). Stochastic multi-objective modeling for optimization of water-food-energy nexus of irrigated agriculture. Adv. Water Resour..

[2] Meng F., Yuan Q., Bellezoni R.A., de Oliveira J.A.P., Cristiano S., Shah A.M., Liu G., Yang Z., Seto K.C. (2023). Quantification of the food-water-energy nexus in urban green and blue infrastructure: a synthesis of the literature. Resour. Conserv. Recycl..

[3] Gleick Peter H. (1994). Water and energy. Annu. Rev. Energy Environ..

[4] Howells M., Rogner H.H. (March. 2014). Water-energy nexus: assessing integrated systems. Nat. Clim. Change.

[5] Stokes J., Horvath A. (2009). Energy and air emission effects of water supply. Environ. Sci. Technol..

[6] Filion Yves R., MacLean Heather L., Asce A.M., Karney Bryan W., Asce M. (2004). Life-cycle energy analysis of a water distribution system. J. Infrastruct. Syst..

[7] Arpke A., Hutzler N. (2008). Domestic water use in the United States. J. Ind. Ecol..

[8] Majid Aman, Cardenes Iliana, Zorn Conrad, Russell Tom, Colquhoun Keith, Bañares-Alcantara René, Hall Jim W. (2020). An analysis of electricity consumption patterns in the water and wastewater sectors in South East England, UK. Water.

[9] Klein G., Krebs M., Hall V., O'Brien T., Blevins B.B. (2005).

[10] Yang L., Zeng S., Chen J., He M., Yang W. (September. 2010). Operational energy performance assessment system of municipal waste water treatment plants. Water Sci. Technol..

[11] Grzegorzek M., Wartalska K., Kaźmierczak B. (2023). Review of water treatment methods with a focus on energy consumption. Int. Commun. Heat Mass Tran..

[12] Cleugh Helen, Stafford Smith Mark, Michael Battaglia and Paul Graham (2011).

[13] Algarni S., Tirth V., Alqahtani T., Alshehery S., Kshirsagar P. (2023). Contribution of renewable energy sources to the environmental impacts and economic benefits for sustainable development. Sustain. Energy Technol. Assessments.

[14] Huei, Ping Li (2019).

[15] Copeland Claudia, Carter Nicole T. (January. 2014).

[16] Vanitha S., Radhika K., Boopathi S., Vasant P., Rodríguez-Aguilar R., Litvinchev I., Marmolejo-Saucedo J. (2023). Human Agro-Energy Optimization for Business and Industry.

[17] Wakeel M., Chen B., Hayat T., Alsaedi A., Ahmad B. (September. 2016). Energy consumption for water use cycles in different countries: a review. Appl. Energy.

[18] Allen P.J., Brennan M.J. (2008). “Energy management systems,” HPAC heating, piping. AirConditioning Eng..

[19] Ruiz G.R., Bandera C.F. (2017). Validation of calibrated energy models: common errors. Energies.

[20] ASHRAE Guideline 14-2014 (2014). Measurement of energy, demand, and water savings. ASHRAE Guidel..

[21] Efficiency Valuation Organization (2016). International performance measurement & verification Protocol. Handb. Financ. Energy Proj..

[22] Keyo (2019). Simple linear regression. https://medium.com/.

[23] Huang Tommy (2018).

[24] Lynn (2021). Rise and fall of machine learning: from neural network to shallow learning. https://www.stockfeel.com.

[25] Wu Chun-Hsin, Ho Jan-Ming, Lee Der-Tsai (2004). Travel-time prediction with support vector regression. IEEE Trans. Intell. Transport. Syst..

[26] Kavitha S., Varuna S., Ramya R. (2016). 2016 Online International Conference on Green Engineering and Technologies (IC-GET).

[27] Atique Sharif (2020). 2020 IEEE Green Technologies Conference (GreenTech).

[28] Phan Quoc-Thang (2022). A novel forecasting model for solar power generation by a deep learning framework with data preprocessing and postprocessing. IEEE Trans. Ind. Appl..

[29] Zhou Haomiao (2016). A new sampling method in particle filter based on Pearson correlation coefficient. Neurocomputing.

[30] Bai-Gang Du, Qi-Liang, Zhou Jun, Guo, Shun-Sheng Kuo, Yi-Bing Li, Zhao, Peng Lei, Wang (2019). A DWT-PCA-LSTM-based water supply forecasting device for water supply companies(C.N. Patent No. 111,079,989A). China National Intellectual Property Administration.

